# Sex affects the response of Wistar rats to polyvinyl pyrrolidone (PVP)-coated silver nanoparticles in an oral 28 days repeated dose toxicity study

**DOI:** 10.1186/s12989-021-00425-y

**Published:** 2021-10-18

**Authors:** Marija Ćurlin, Rinea Barbir, Sanja Dabelić, Marija Ljubojević, Walter Goessler, Vedran Micek, Irena Žuntar, Mirela Pavić, Lucija Božičević, Ivan Pavičić, Ivana Vinković Vrček

**Affiliations:** 1 School of Medicine, University of Zagreb, Šalata 3, 10 000 Zagreb, Croatia; 2 Institute for Medical Research and Occupational Health, Ksaverska cesta 2, 10 000 Zagreb, Croatia; 3 Faculty of Pharmacy and Biochemistry, University of Zagreb, Ante Kovačića 1, 10 000 Zagreb, Croatia; 4 Faculty of Veterinary Medicine, University of Zagreb, Heinzelova 55, 10 000 Zagreb, Croatia; 5 Institute of Chemistry, University of Graz, Universitätsplatz 1/1, 8 010 Graz, Austria

**Keywords:** Accumulation, Toxicity, Oxidative stress, Metallothionein, Wistar rat

## Abstract

**Background:**

Silver nanoparticles (AgNPs) are widely used in biomedicine due to their strong antimicrobial, antifungal, and antiviral activities. Concerns about their possible negative impacts on human and environmental health directed many researchers towards the assessment of the safety and toxicity of AgNPs in both in vitro and in vivo settings. A growing body of scientific information confirms that the biodistribution of AgNPs and their toxic effects vary depending on the particle size, coating, and dose as well as on the route of administration and duration of exposure. This study aimed to clarify the sex-related differences in the outcomes of oral 28 days repeated dose exposure to AgNPs.

**Methods:**

Wistar rats of both sexes were gavaged daily using low doses (0.1 and 1 mg Ag/kg b.w.) of polyvinylpyrrolidone (PVP)-coated small-sized (10 nm) AgNPs. After exposure, blood and organs of all rats were analysed through biodistribution and accumulation of Ag, whereas the state of the liver and kidneys was evaluated by the levels of reactive oxygen species (ROS) and glutathione (GSH), catalase (CAT) activity, superoxide dismutase (SOD) and glutathione peroxidase (GPx), expression of metallothionein (Mt) genes and levels of Mt proteins.

**Results:**

In all animals, changes in oxidative stress markers and blood parameters were observed indicating the toxicity of AgNPs applied orally even at low doses. Sex-related differences were noticed in all assessed parameters. While female rats eliminated AgNPs from the liver and kidneys more efficiently than males when treated with low doses, the opposite was observed for animals treated with higher doses of AgNPs. Female Wistar rats exposed to 1 mg PVP-coated AgNPs/kg b.w. accumulated two to three times more silver in the blood, liver, kidney and hearth than males, while the accumulation in most organs of digestive tract was more than ten times higher compared to males. Oxidative stress responses in the organs of males, except the liver of males treated with high doses, were less intense than in the organs of females. However, both Mt genes and Mt protein expression were significantly reduced after treatment in the liver and kidneys of males, while they remained unchanged in females.

**Conclusions:**

Observed toxicity effects of AgNPs in Wistar rats revealed sex-related differences in response to an oral 28 days repeated exposure.

**Supplementary Information:**

The online version contains supplementary material available at 10.1186/s12989-021-00425-y.

## Background

Silver nanoparticles (AgNPs) are one of the most exploited and investigated engineered nanomaterials. They are widely utilized in medicine, cosmetics, textile engineering and electronics due to their strong antimicrobial, antifungal, and antiviral activity [1–3]. However, the question about their safety and potential adverse effects on human and environmental health is still prevailing [4–6]. Their toxic effects are not yet fully understood, as well as an exact mechanism of their bactericidal action [7, 8]. A growing base of evidence suggests that, at the cellular level, the adverse outcomes of exposure to AgNPs depend on the uptake of AgNPs by cells, cell membrane damage, and activation of signalling pathways by interactions with membrane proteins which leads to inhibition of cell proliferation and apoptosis [9]. Internalized particles may cause mitochondrial damage and dysfunction, induce reactive oxygen species (ROS) generation, cause DNA damage, protein carbonylation, and membrane oxidation, which all inevitably lead to damage of proteins and nucleic acids inside the cell, and, finally, inhibition of cell proliferation [9]. The mechanism of adverse AgNP effects depends on many factors like particle size, surface coating, and solubility of the AgNPs [10–17]. Small-sized AgNPs (≤ 10 nm) have stronger antibacterial and cytotoxic effects [11, 12], along with better biodistribution and bioaccumulation compared to larger particles [14]. The surface coating may be designed to provide long-term colloidal stability and to enhance or prevent the release of Ag^+^ from AgNPs depending on the intended use of AgNPs [10, 15].

Adverse outcomes and biodistribution pattern of AgNPs depend not only on particle characteristics, but also on the duration of exposure, applied dose, and the route of administration [16–23]. The orally ingested AgNPs pass through the gastrointestinal tract, enter the blood circulation being distributed to different organs including the liver and kidneys [16, 19, 24, 25]. At each point of this route, AgNPs may induce different histopathological and biochemical changes depending on the dose, particle size, and surface coating [21, 24, 26–31]. It should be highlighted that different transformation of AgNPs may be expected during their journey through different body compartments. Indeed, we have recently demonstrated that AgNPs may not only agglomerate, but also degrade and dissolve to ionic forms, which can again reconstruct to NPs or precipitate as Ag sulphides or chlorides [26]. Only few studies demonstrated accumulation of AgNPs in different organs using techniques like transmission electron microscopy (TEM) or single particle inductively coupled plasma mass spectrometer (ICP-MS) [16, 20, 24, 29], while most other studies just evaluated total Ag content in different organs or tissues by ICP-MS [18, 19, 28].

Since proposed adverse outcomes of exposure to AgNPs and other metallic NPs involves ROS generation and oxidative stress induction [32–37], several studies have investigated oxidative and inflammatory effects after oral exposure of rodents to high doses of AgNPs [14, 38, 39]. Changes in these parameters were observed even at low AgNPs doses in both acute and sub-acute/sub-chronic exposure experiments [28, 40–42]. For example, low doses of AgNPs (1 mg Ag/kg) after 14 days of oral exposure induced a significant increase in ROS and inflammatory markers, and depletion of antioxidant enzyme status in erythrocytes with evidence of hepatic and renal toxicity in mice [43]. Recent study evaluated changes in expression of metallothioneins (Mt), which are cysteine rich protein in dumbbell shape conformation with two domains that bind 7 divalent or even more monovalent metal ions in non-cooperative fashion [44]. Thus, mammalian inducible Mt 1 and Mt 2 respond to various stimuli including essential and toxic metals they bind like silver [45]. Recent in vitro experiments showed AgNP induction of Mt in mammalian neural cells [45]. Only few studies investigated oxidative toxicity of low AgNPs doses orally applied to rats but reported no functional or histopathological changes except the ultrastructural disturbances in myelin sheaths [28, 40, 41]. Nevertheless, the subtle changes in redox balance at the cellular level may later result in serious adverse outcomes [46].

Sex-related differences in the toxic effects of metal NPs, although being subtle and in many cases insignificant, are still intriguing researchers’ interest [47–49]. Sex-related differences in the oxidative toxicity of silver nanoparticles, particularly of low AgNP doses are scarcely documented. A study of orally exposed mice to AgNPs did not show significant differences in toxic effects of AgNP on liver and some hematological parameters between males and females [27]. Our recent comprehensive study of sex-related differences in oxidative stress parameters in mice treated with AgNPs intraperitoneally during 21 days revealed sex-related differences in oxidative stress parameters in the liver, kidneys, brain and lungs [50], while our systematic investigation of the protein corona impact on the biodistribution and toxicity effects of AgNPs given orally to rats detected differences in the response of male compared to female rats [42]. Other studies were mostly focused on the sex-related differences in the biodistribution and accumulation of AgNPs [20, 51–53]. Literature search of the Web of Science and PubMed databases (performed in February 2021 by using keywords “silver AND nano*” AND “in vivo”) revealed no systematic in vivo study on the impact of sex on oxidative and inflammatory response after a low dose oral exposure of rats to small AgNPs (see Table 1).

**Table 1:** Reported data on sex-related toxicity and biodistribution of AgNPs in rats via sub-chronic oral exposure

Animal	AgNPs properties (size, coating)	Dosage and exposure time	Sex-related ADME and toxicity effects	Reference
Sprague–Dawley rats	60 nm, coated with carboxymethyl-cellulose	30, 300, 1000 mg/kg b. w./day; 28 days oral administration	Twofold higher Ag accumulation in females	[21]
Fischer 344 rats	60 nm, coated with carboxymethyl-cellulose	30, 125, 500 mg/kg b.w./day; 90 days oral administration	Higher Ag accumulation in kidneys of females	[51]
Fischer 344 rats	56 nm, coated with carboxymethyl-cellulose	30, 125, 500 mg/kg b.w./day; 90 days oral administration	Twofold higher Ag accumulation in kidneys of females; decrease in the bodyweight of males	[53]
Sprague–Dawley rats	10, 75, and 110 nm, citrate-coated	10 mg/kg b.w.; single oral administration 9, 18, 36 mg/kg b.w./day; daily oral gavage for 13 weeks	Higher Ag accumulation in kidney, liver, jejunum, and colon of females	[20]
Sprague–Dawley rats	10, 75, and 110 nm, citrate-coated	9, 18, 36 mg/kg b.w./day; daily oral gavage for 13 weeks	Sex-specific effects more prominent for the gut-associated immune responses	[54]
Sprague–Dawley rats	10 and 110 nm, citrate-coated	9 mg/kg b.w./day	Upregulated tight junction genes expression and TNF-α in females	[55]

With this study, we aimed to fill the knowledge gaps on potential sex-related differences in biodistribution and oxidative stress induction during the 28 days oral application of polyvinylpyrrolidone (PVP)-coated AgNPs at doses of 0.1 and 1 mg per kg of body weight (b.w.). The PVP was selected as the most frequently used surface coating material for metallic NPs [13]. Obtained results bring a valuable contribution to a reliable evidence base supporting the assumption that subtle, but pertinent changes at the cellular and organ level appear because of the exposure to low AgNPs doses. The knowledge of the sex-related differences, even at the cellular level, highlights the importance of the sex-related toxicology approach.

## Results

### Physicochemical characteristics of PVP-AgNPs

**Fig. 1:**
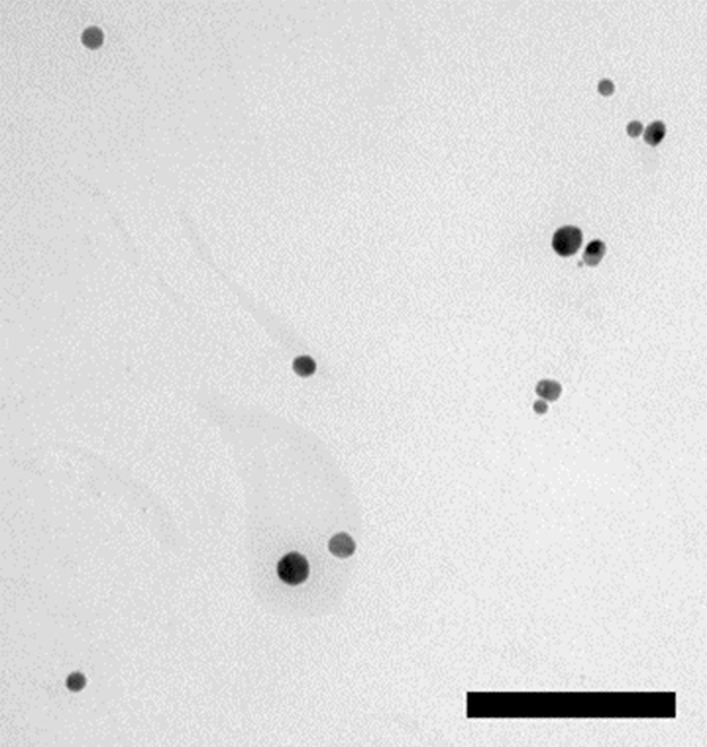
Transmission electron micrograph of PVP-coated silver nanoparticles in ultrapure water. The scale bar is 100 nm

Visualization by TEM confirmed the spherical shape and size of the particles as intended by synthesis protocol (Fig. 1). The primary size of AgNPs was 8.6 ± 1.9 nm as determined by TEM, and dynamic light scattering (DLS) showed their monomodal size distribution with the hydrodynamic size of 12.1 ± 3.4 nm. The electrophoretic light scattering (ELS) evaluation revealed a slightly negative ζ potential value of − 12.6 ± 1.4 mV. Quantification of soluble Ag fraction after the 1-h incubation of AgNPs in ultrapure water showed that PVP-AgNPs released 4.3% of Ag^+^ ions. Concerning the doses administered to animals (0.1 and 1 mg Ag/kg b.w.), it may be assumed that the soluble Ag levels in these doses were around 3 and 30 μg Ag/kg b.w., respectively. Thus, only main physico-chemical characteristics of AgNPs are presented here, while detailed evaluation of their colloidal stability and behaviour under different biological conditions relevant for in vivo studies are described elsewhere [26].

### Biodistribution and bioaccumulation of silver

As expected, the Ag content in all organs was higher in the groups of animals treated with 1 mg Ag/kg b.w. of AgNPs (HD groups) (Table 2). In the groups of animals treated with 0.1 mg Ag/kg b.w. of AgNPs (LD groups), the Ag accumulation was not significant (at *p* < 0.05) in any tested organ (Table 2).

**Table 2:** The Ag content (in mg per kg of tissue) in heart, brain, liver and kidney, blood and reproductive organs and gastrointestinal tract of male and female rats after administration of LD and HD of PVP-AgNPs

Tissue	Males			Females		
	Ctl	LD	HD	Ctl	LD	HD
Blood	0.01 ± 0.00	5.63 ± 2.11	19.02 ± 1.12*	0.01 ± 0.00	3.62 ± 0.81	49.31 ± 7.55^§^
Liver	0.03 ± 0.02	0.10 ± 0.08	0.20 ± 0.04	0.01 ± 0.00	0.05 ± 0.01	0.41 ± 0.05*^§^
Kidney	0.02 ± 0.00	0.08 ± 0.03	0.23 ± 0.4	0.02 ± 0.00	0.06 ± 0.02	0.75 ± 0.04^§^
Heart	0.01 ± 0.00	0.01 ± 0.00	0.02 ± 0.00	0.01 ± 0.00	0.01 ± 0.00	0.05 ± 0.00^§^
Brain	0.01 ± 0.00	0.16 ± 0.11	0.25 ± 0.01	0.01 ± 0.00	0.04 ± 0.01	0.28 ± 0.02*
Stomach	0.01 ± 0.00	0.03 ± 0.01	0.15 ± 0.04*	0.02 ± 0.03	0.11 ± 0.00	2.26 ± 1.76*^§^
Duodenum	0.01 ± 0.00	0.03 ± 0.01	0.05 ± 0.01	0.01 ± 0.00	0.04 ± 0.03	0.61 ± 0.47*^§^
Jejunum	0.01 ± 0.00	0.07 ± 0.05	0.16 ± 0.07	0.01 ± 0.00	0.01 ± 0.00	4.12 ± 3.64
Ileum	0.01 ± 0.00	0.03 ± 0.01	0.06 ± 0.03	0.01 ± 0.00	0.02 ± 0.00	0.09 ± 0.03
Cecum	0.01 ± 0.00	0.16 ± 0.08	0.68 ± 0.40	0.01 ± 0.00	0.05 ± 0.03	5.15 ± 1.15
Colon	0.01 ± 0.00	0.52 ± 0.47	1.42 ± 0.57	0.01 ± 0.00	0.02 ± 0.01^§^	3.37 ± 0.32*
Testis	0.01 ± 0.00	0.04 ± 0.02	0.13 ± 0.01*	n.a	n.a	n.a
Epididymis	0.01 ± 0.00	0.18 ± 0.17	0.27 ± 0.04	n.a	n.a	n.a
Ovary	n.a	n.a	n.a	0.01 ± 0.00	0.01 ± 0.00	0.13 ± 0.03

*p < 0.05 and **p < 0.001 between controls (Ctl) and treated animals (for both LD and HD groups), ^§^*p* < 0.05 between males and females

**Fig. 2:**
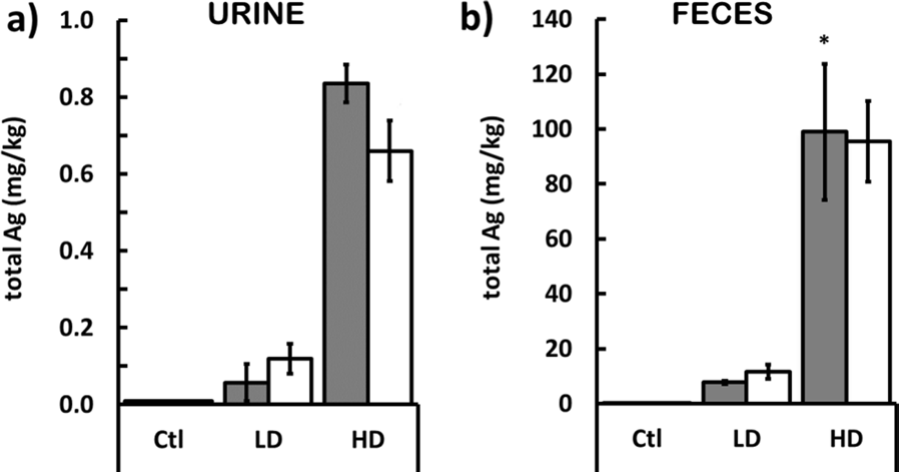
The Ag content in **a** urine and **b** feces of male (gray columns) and female (white columns) rats after administration of LD and HD of PVP-AgNPs. Error bars: SD. *p < 0.05 between controls (Ctl) and treated animals (for both LD and HD groups)

In the HD group, Ag accumulation in organs of females was almost regularly significantly higher than in males, except in the brain where it was equal. In the blood, liver, kidney and heart of females, the accumulation was two to three times higher than in males, while in most organs of digestive tract, it was more than ten times higher. In males, the increase was moderate, significant only in the blood, stomach and testis (at *p* < 0.05), and disproportional to the increase of the applied dose when comparing males from LD and HD groups (Table 2). An exception was an increase in Ag content in urine and feces showing a dose-depentent elimination of Ag (Fig. 2).

### Sex-related oxidative stress response in kidney and liver

**Fig. 3:**
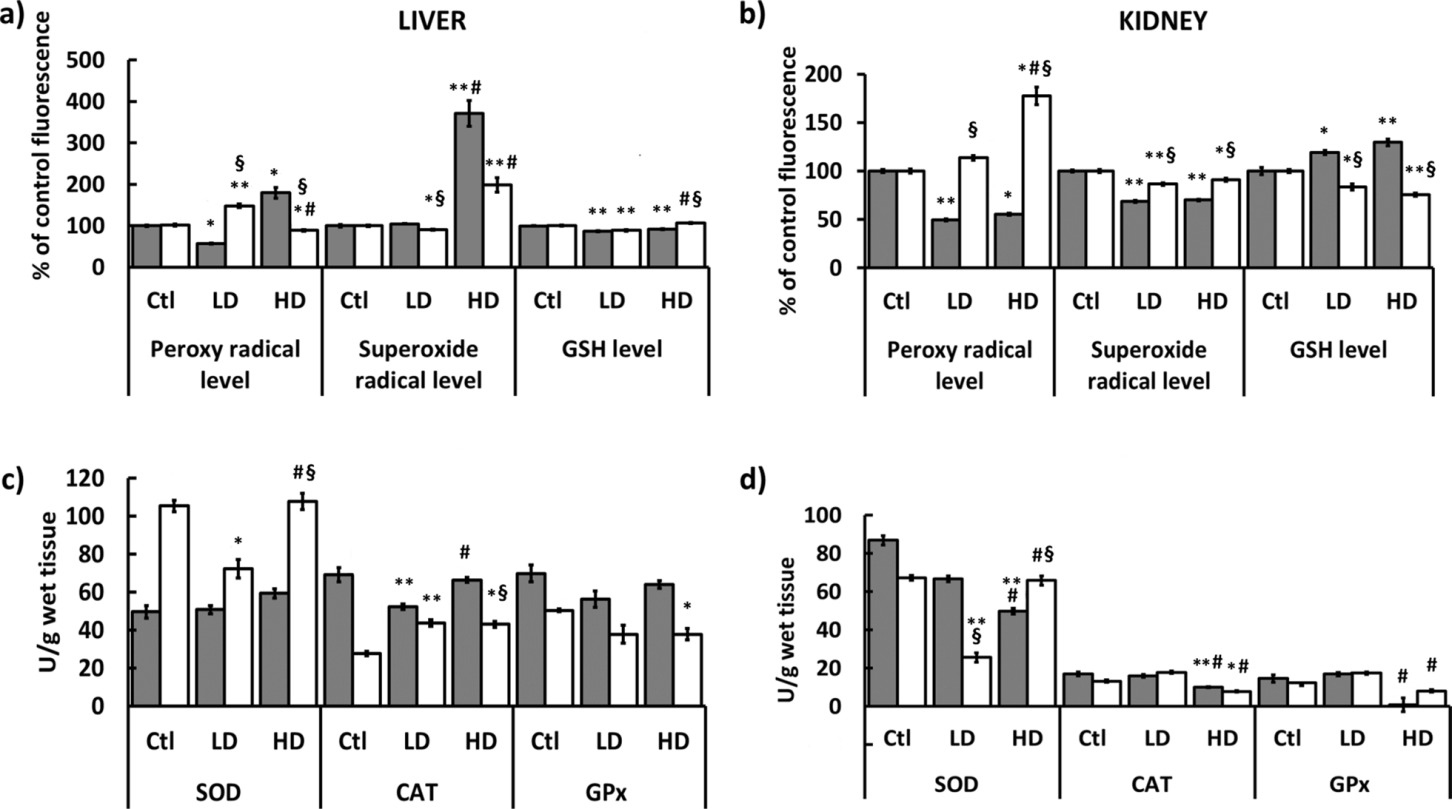
Parameters measured in the liver and kidneys of male (gray columns) and female (white columns) rats after administration of LD and HD of PVP-AgNPs. **a**, **b** Levels of peroxy radical, levels of superoxide radical and levels of glutathione given as % of fluorescence compared to the control. **c**, **d** SOD activity, CAT activity, and GPx activity given as units of enzyme activity per gram of wet tissue. Error bars: SD. *p < 0.05 and **p < 0.001 between controls (Ctl) and treated animals, ^#^*p* < 0.05 between LD and HD groups of the same sex and ^§^*p* < 0.05 between males and females

Levels of peroxy radicals in the liver changed significantly after exposure to AgNPs, but the pattern of the change was quite different in males and females (Fig. 3a). In LD group males, this level was reduced, whereas a significant increase was observed in the HD group, almost 2-times higher than in the control. On the contrary, the LD of AgNPs increased the levels of peroxy radicals in females, whereas the HD decreased it to a level lower than observed in the control group. Levels of peroxy radicals in male kidneys were reduced to a half after the exposure to AgNPs in LD and HD groups, while a significant and dose-dependent increase was observed in females (Fig. 3b).

Levels of superoxide radicals decreased significantly in the liver of females after exposure to the low dose of AgNPs, but statistically significant increase was observed in both male and female HD groups (Fig. 3a). The increase in the liver of HD males was two times greater compared to females. In kidneys, the level of superoxide radicals significantly decreased in animals of both sexes from both LD and HD groups compared to controls. The decrease in the male kidneys was greater by 20% than in the kidneys of females (Fig. 3b).

The changes of glutathione (GSH) levels, although statistically significant, were subtle in the liver of all treated groups (Fig. 3a). In the liver of males, GSH was reduced by cca 10% after both LD and HD treatment. In the liver of females, the LD exposure to AgNPs reduced, while the HD exposure increased the GSH level. Female and male rats showed opposite results in the GSH levels in kidneys (Fig. 3b). In kidneys of males, GSH was increased in a dose-dependent manner, while the dose-dependent decrease was observed in kidneys of females.

In the liver of males, superoxide dismutase (SOD) activity remained the same after exposure to LD AgNPs and slightly increased after exposure to HD as compared to controls (Fig. 3c). In the kidneys of males, SOD activity was reduced in a dose-dependent manner in both LD and HD groups (Fig. 3d). The liver and kidneys of females showed the same pattern of changes in SOD activity: the LD AgNP reduced it and the HD raised it back to control levels.

In the liver of males, the activity of catalase (CAT) was reduced in the LD group, but no significant change was observed in the HD compared to the control group (Fig. 3c). In kidneys of males, a significant change in CAT activity was observed only after exposure to HD AgNPs, as it was decreased when compared to the control (Fig. 3d). In the liver of females, CAT activity decreased after exposure to both LD and HD AgNPs (Fig. 3c). In kidneys of females, CAT activity was increased in the LD group but was significantly decreased in the HD group compared to the control (Fig. 3d).

No significant change was observed in the activity of glutathione peroxidase (GPx) in the liver of both sexes with an exception of the female HD group where the activity was significantly decreased (Fig. 3c). In the kidneys of males, a non-significant reduction of GPx was measured in the HD group, as well as in the female HD group (Fig. 3d). In the female LD group, GPx activity was increased.

### Metallothionein genes and proteins expression

**Fig. 4:**
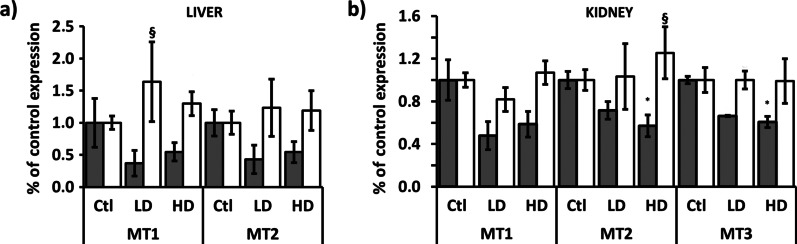
mRNA expression of Mt genes. **a** Mt1a and Mt2a in the liver and **b** Mt1a, Mt2a, and Mt3 in kidneys of male (gray columns) and female (white columns)) rats after administration of LD and HD of PVP-AgNPs. mRNA expression levels are presented as relative values compared to control samples. Error bars: SD. *p < 0.05 between controls (Ctl) and treated animals and ^§^*p* < 0.05 between males and females

Quantitative TaqMan-based real-time PCR was performed to investigate the effect of AgNPs treatment on the relative mRNA levels of three Mt isoforms—Mt1a, Mt2a, and Mt3 in the liver and kidneys of male and female animals. The results are presented in Fig. 4.

Reduction of the mRNA levels of both Mt1a and Mt2a by 35% to 80% was observed in the liver of all of the male rats from the LD group. The HD treatment provoked a similar effect—the rate of inhibition ranged from 30 to 70% for both Mt1a and Mt2a isoforms in the liver of male rats (Fig. 4a). Though female rats showed more pronounced inter-individual differences as a response to AgNPs treatment, the observed changes in the mRNA expression levels for both Mt1a and Mt2a were biologically insignificant, and inside the deviation caused by the limitation of the experimental procedure. Namely, neither LD nor HD caused changes in the Mt1a and Mt2a gene expression in the liver of females. In the liver, regardless of the sex, the levels of Mt3 were practically undetectable.

A decrease in the mRNA level for all Mt isoforms was observed in the kidneys of male rats in both groups (Fig. 4b). The LD treatment caused a reduction by 30–70% for Mt1a, 23–63% for Mt2a, and around 35% for Mt3 in kidneys of male animals. The higher dose showed a similar effect in male kidneys—inhibition ranged from 30 to 62% for Mt1a, 30–54% for Mt2a, and 35–50% for Mt3. However, females did not show any changes in mRNA levels of all tested Mt isoforms for kidneys (Fig. 4b).

**Fig. 5:**
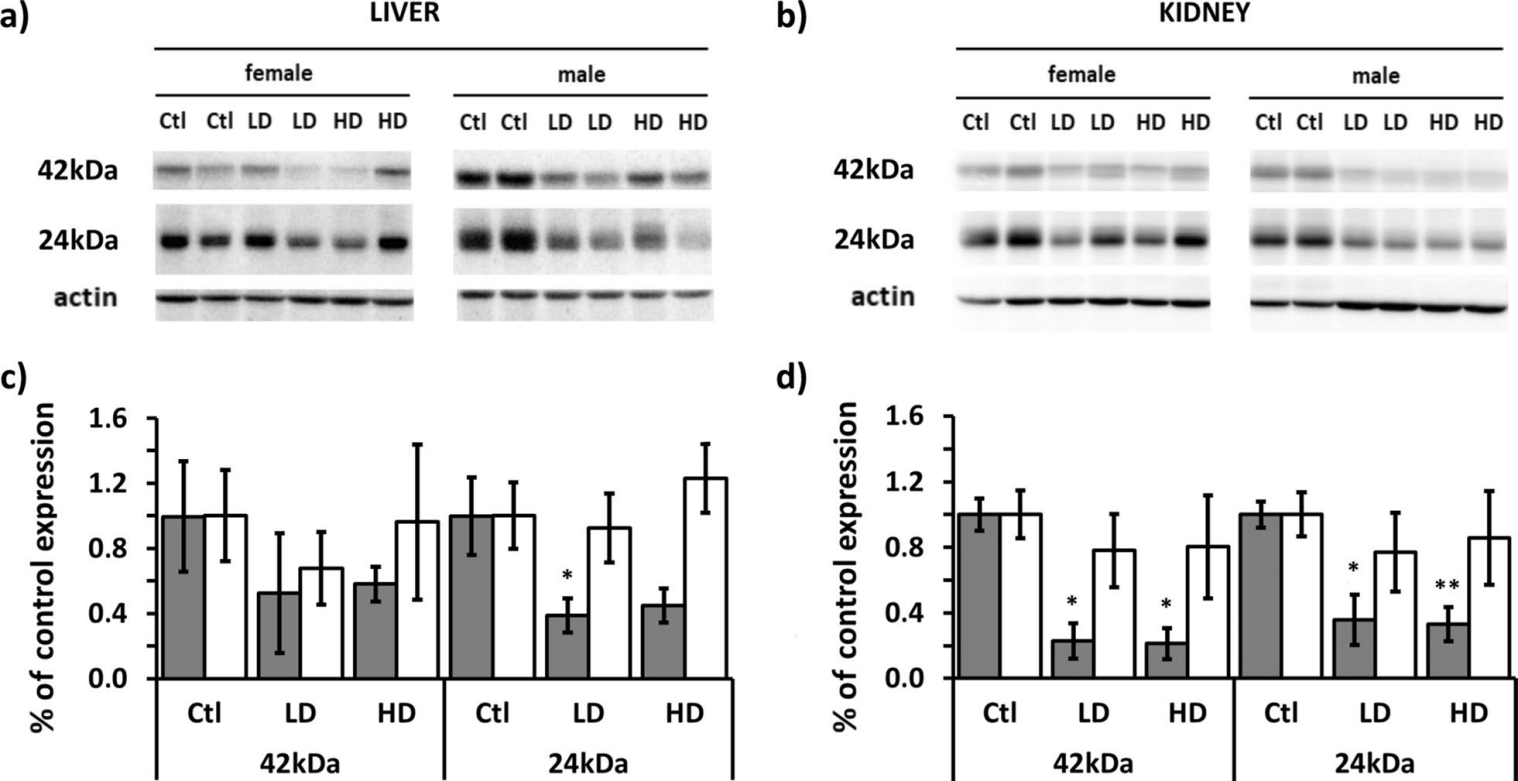
Protein expression of two multimeric forms (~ 42 kDa and ~ 24 kDa) of metallothionein MT1/2 proteins, in the liver and kidneys of male (gray columns) and female (white columns) rats after administration of LD and HD of PVP-AgNPs. **a**, **b** Represent Western blot images for liver and kidneys, respectively, while **c**, **d** diagrams show protein expression levels in liver and kidneys, respectively, presented as relative values compared to control samples. Error bars: SD. *p < 0.05 and **p < 0.001 between controls (Ctl) and treated animals

Expression of Mt proteins in kidney and liver homogenates was measured by chemiluminescent-western blot analysis. Immunogen for used primary mouse monoclonal anti-metallothionein antibody is self-polymerized Mt1 and Mt2, and two self-polymerized forms of approximately 42 kDa and 24 kDa were detected. Though some tendency for reduction of Mt proteins expression was observed in females, the inter-individual differences were quite large and resulted in no statistically significant impact of AgNPs treatment. On the contrary, among male animals, both detected polymerized forms of Mt showed a similar pattern of response to LD and HD treatment—the inhibitory effect of approximately 50% in liver and 70% in kidney, was detected (Fig. 5).

### Blood biochemistry and hematological parameters

**Fig. 6:**
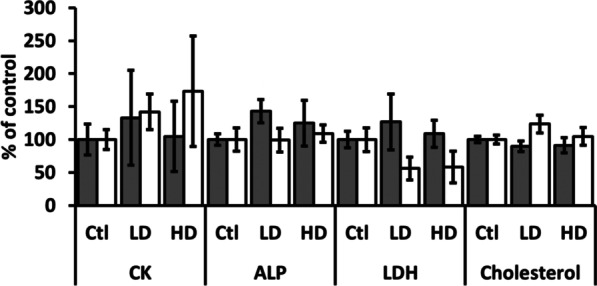
Biochemical blood parameters of male (gray columns) and female (white columns) rats after administration of LD and HD of PVP-AgNPs. All results are expressed as relative values compared to the control samples. Error bars: SD

**Fig. 7:**
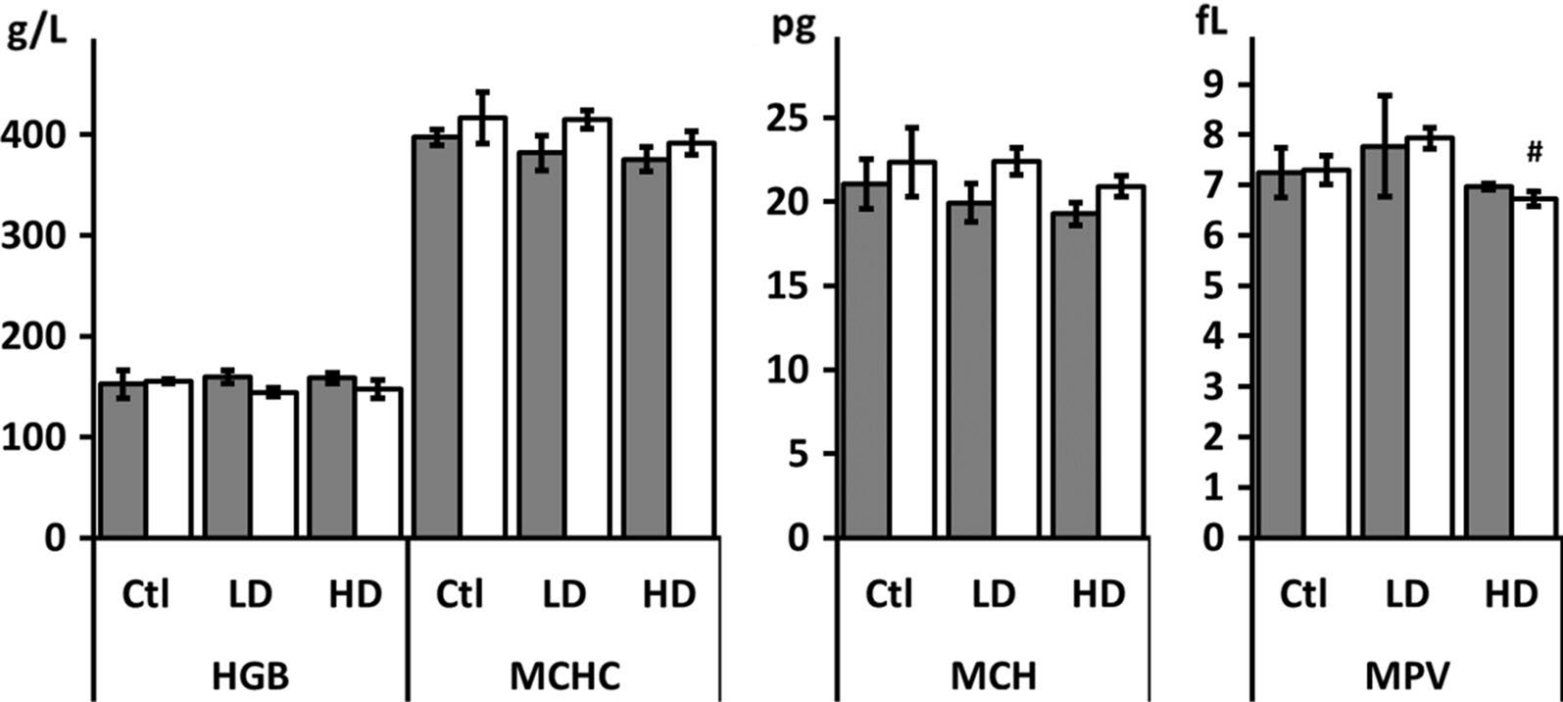
Hematological parameters of male (gray columns) and female (white columns) rats after administration of LD and HD of PVP-AgNPs. Error bars: SD. ^#^*p* < 0.05 between LD and HD groups of the same sex

Biochemical analysis of serum in control, LD, and HD groups did not show statistically significant changes in any biochemistry and hematological parameter (Additional file 1). Although insignificant, the results obtained for creatine kinase (CK), alkaline phosphatase (ALP), lactate dehydrogenase (LDH) and cholesterol levels, as well as for hemoglobin (HGB), mean corpuscular hemoglobin (MCH), mean corpuscular hemoglobin concentration (MCHC) and mean platelet volume (MPV) indicate some changes, so they are described and graphically demonstrated in Figs. 6 and 7.

The activity of CK was non-significantly elevated in male rats exposed to low AgNP dose, while females showed a dose-dependent CK increase. In females from the LD group, CK activity was increased. However, in females from the HD group, CK activity was increased almost twofold compared to controls, but the inter-individual differences were quite large and resulted in no statistically significant impact of AgNPs treatment. ALP was increased in males from both groups. LDH was reduced in females exposed to both low and high AgNP doses. Cholesterol was slightly increased in females exposed to low but not in females exposed to high AgNP dose.

**Fig. 8:**
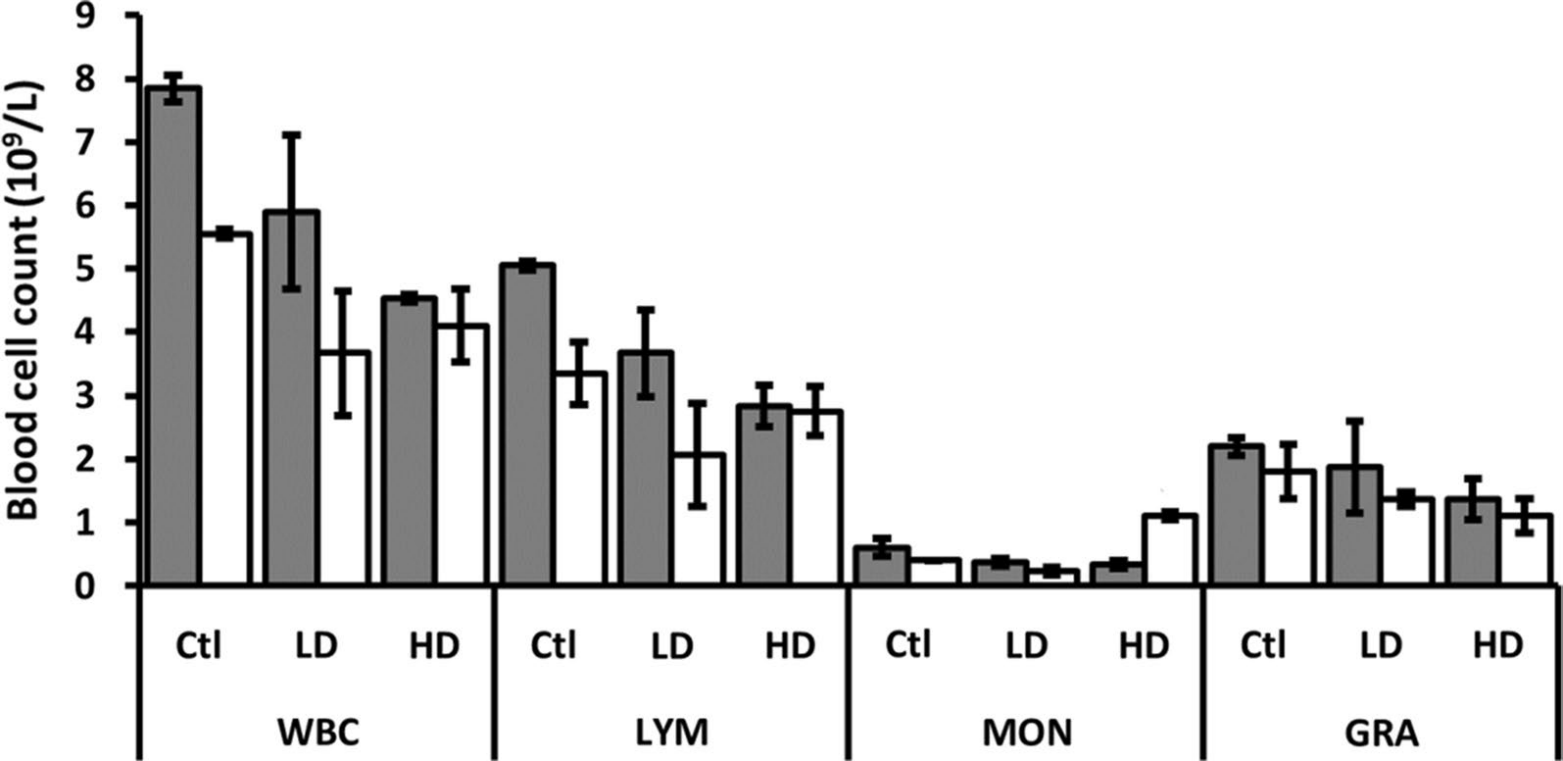
Differential blood cell count of male (gray columns) and female (white columns)) rats after administration of LD and HD of PVP-AgNPs. Error bars: SD

The hemoglobin (HGB) level decreased in the females exposed to low AgNP dose, while mean corpuscular hemoglobin (MCH) and mean corpuscular hemoglobin concentration (MCHC) showed a decrease in females exposed to high AgNP dose compared to control animals (Fig. 7). The mean platelet volume (MPV) was reduced only in female rats exposed to HD AgNP (Fig. 7), while no changes were observed for platelets (PLT), red blood cells (RBC), red cell distribution width (RDW), hematocrit (HCT) and mean corpuscular volume (MCV) (Additional file 1). The white blood cell (WBC) and monocyte (MON) counts were reduced in females of both LD and HD groups compared to controls (Fig. 8). In males, the WBC numbers were also reduced, but the MON number increased in a dose-dependent manner. The granulocyte (GRA) number in males was reduced in rats exposed to HD AgNP compared to controls, while no changes were observed in females. The lymphocyte (LYM) count was reduced only in male rats in a dose-dependent manner (Fig. 8).

## Discussion

This study aimed to observe differences in the response of male and female Wistar rats to oral exposure to LD (0.1 mg/kg b.w.) and HD (1 mg/kg b.w.) of PVP-coated AgNPs for 28 days. The selected experimental settings resembled the actual concentrations and conditions in which humans and animals are exposed to AgNPs from drugs and food and drink containers [56]. The precise information on sex-related responses to AgNPs could be of great value for the assessment of particular health risks of each sex. During 28 days oral exposure to AgNPs, the general condition and clinical symptoms of experimental animals were carefully monitored. There were no changes in body weight, hair condition, breathing, behaviour and movement in AgNP-treated compared to control animals.

Toxic effects of AgNPs were assessed by analysis of oxidative stress markers (levels of ROS and GSH and activities of CAT, SOD, and GPx), by determination of gene and protein expression of Mt isoforms in the liver and kidney, and by blood cell count and biochemical analysis of serum. It should be noted that obtained results could not be attributed to a single action of AgNPs since the assessment of their colloidal stability showed the release of 4.3% of Ag ions in ultrapure water indicating that the animals, receiving either low or high doses of AgNPs, were exposed to 3 and 30 μg ionic Ag per kg b.w., respectively. Even higher Ag^+^ release can be expected in the gastric and lysosomal fluid after ingestion of AgNPs. This information should be taken into account since one of the proposed mechanisms of AgNP toxicity includes the toxic effects of Ag^+^ ions released from their surface [57, 58]. However, it cannot be distinguished what portion of the AgNP toxicity originates from the ionic form and what portion from the nanoparticulate form as AgNPs may encounter diverse biotransformation patterns on their way throughout the body [24, 59].

In order to be able to faithfully interpret AgNP toxic effects, Ag biodistribution analysis and determination of Ag accumulation in the treated organs were performed. The results of this study agree quite well with previous investigations that showed Ag accumulation in the gastrointestinal system, blood, liver, kidneys, testis, brain, urine, and feces [18–21, 24, 28, 40, 60]. We found additional Ag accumulation in ovaries of females, and epididymis of male rats. Moreover, this study confirmed that females accumulate a higher amount of Ag, not only in the jejunum, colon, liver, and kidneys, but also in all tested organs as already observed by other research groups (see Table 1) who evaluated AgNP biodistribution in rodent models [19–21]. Surprisingly, these findings were valid only for animals treated with the higher dose (1 mg Ag/kg b.w.), whereas the opposite effect was frequently noted in animals treated with the lower AgNP dose (0.1 mg Ag/kg b.w.) as can be seen in Table 2. Due to pronounced inter-individual differences, the average Ag accumulation values in males were not statistically significant, but are still a warning sign that low AgNP doses may have different distribution between males and females, and hence different toxic effect than high doses tested in previous studies [20, 21, 54, 55].

As a consequence of the accumulation of Ag, either in the nanoparticulate or ionic form, the oxidative stress response can be expected. Sex-related differences in oxidative stress have been reported for humans and experimental animals, showing a higher level of oxidative stress markers and greater antioxidant potential in females over males [61–65]. Our study confirmed that female rats have higher levels of ROS under physiological conditions (control animals data in Additional file 1). After exposure to AgNPs, differences in the expression and/or activities of antioxidant enzymes were observed between males and females (Table 2). In both treated female groups, the liver and kidneys responded to the AgNPs by increasing ROS indicating induction of oxidative stress which may lead to DNA damage, cytotoxicity, and apoptosis (Table 2) [25, 33, 35, 66]. In males, only the liver in the HD group showed a notable increase in oxidative stress markers. Males treated with low AgNPs dose, despite a less efficient Ag elimination compared to females, did not show signs of oxidative stress (ROS were below or equal to control levels). The GSH, the first line of the non-enzymatic defence system against oxidative stress was decreased in the female kidney and male liver, amplifying the AgNP-induced oxidative stress [67, 68].

In addition to the above-mentioned parameters, MTs, intercellular cysteine-rich, metal-binding proteins classified into four isoform groups MT1–MT4 [69], were also analysed as they function as non-enzymatic antioxidants against ROS and are inducible by many transition metals and by oxidative stress [70, 71]. However, Mts showed a significant reduction of their gene expression and protein concentrations in all treated male organs. Quite unexpectedly, subchronic exposure to LD and HD of AgNPs decreased abundance of Mt on the level of mRNA and protein in males, but not in females. Mt role in sequestering ROS and keep metal guarded in the cytosol from unwanted binding on target proteins may be exhausted through 28 days exposure. However, turnover of Mt is established through different mechanisms, one being ROS scavenging and dissolved Ag ion binding while other may be opsonization of AgNPs in endosomal-lysosomal compartment due to strong Ag binding on Mt during prolonged time [9].

**Fig. 9:**
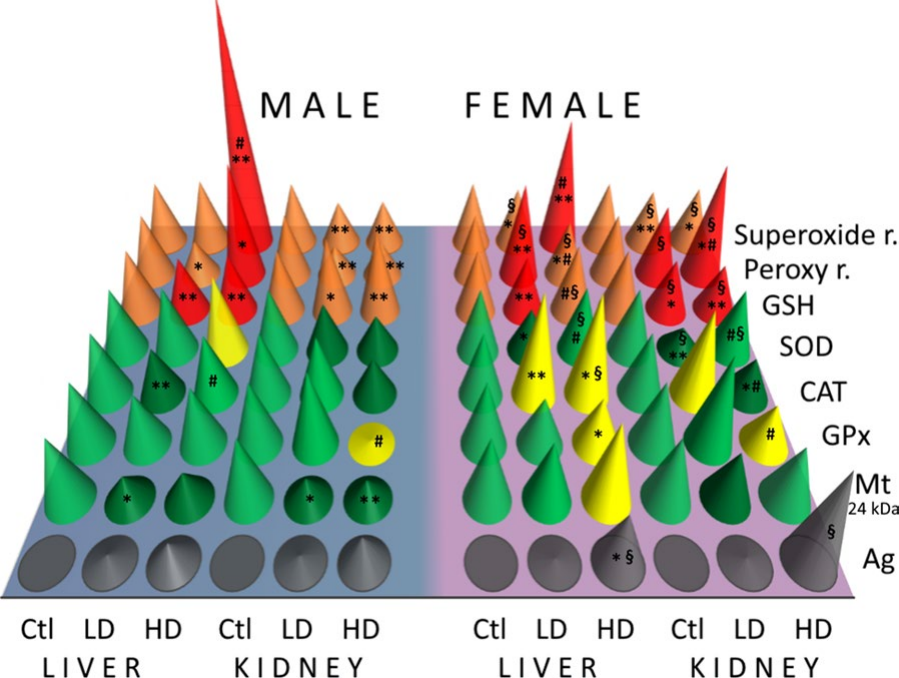
Illustration of relative differences in the reaction of male and female rats to LD and HD of AgNPs. Orange-oxidative stress markers corresponding to no oxidative stress; red-oxidative stress markers indicating oxidative stress; light green–antioxidant levels showing no oxidative stress response; yellow-antioxidant levels showing oxidative stress response; dark green-antioxidant levels showing suppression of oxidative stress response; gray-silver accumulation; blue-male rats; purple-female rats. Cone heights represent values of the measured parameters relative to control values expressed in percentage. All control values are 100%, except the control values for the Ag content which was equal to zero. Ag content in the treated rats is expressed as a difference between sample and control values expressed as a percentage to the arbitrary set 0.5 μg per g of tissue. *p < 0.05 and **p < 0.001 between controls (Ctl) and treated animals, ^#^*p* < 0.05 between LD and HD groups of the same sex and ^§^*p* < 0.05 between males and females

The deficiency of both antioxidants, GSH and MT, may be explained by the suppressing action of AgNPs that directly bind to their thiol groups [68]. In female animals, ROS levels were higher as compared to males, resulting in a stronger induction of MTs and less pronounced direct inhibitory AgNP effect. GSH and MT levels are associated with the availability of Zn in the cell and with its role in the inhibition of inflammation and apoptosis, so a decrease in GSH and MTs may increase both processes in the affected organs [71–73]. Therefore, AgNPs may lead to cell damage not only by inducing excessive ROS generation, but also by several other mechanisms, like the interruption of oxidative stress protection or deregulation of inhibition of apoptosis [66]. Antioxidant enzymes may also be affected by AgNPs in several ways. Generation of excessive ROS induces expression and activation of antioxidant enzymes, but damaging and apoptotic AgNP effects may reduce their activity and ability to defend the cells against oxidative stress [74, 75]. In our study, both kidney and liver from all treated groups showed a reduction in the activity of SOD or CAT or both of them except in livers from HD groups where superoxide radicals levels were extremely high. This indicates the direct damaging effect of AgNPs on protein production and activity. In males from the HD group where kidneys did not show any sign of oxidative stress, the activities of antioxidant enzymes were even more suppressed than in the LD group, which is in agreement with a dose-dependent direct inhibitory interaction of AgNPs with enzymes. Hence, considering the results of oxidative stress markers, and the expression and activity of antioxidant enzymes, all statistically significant aberrations from normal redox balance may be assumed as toxic effects of AgNPs, either as a result of ROS overgeneration or as a direct AgNP or Ag^+^ damaging or inhibitory effect. The correlative results in the male and female rats do not show consistent sex differences in the sensitivity and response to oxidative stress. Nevertheless, a substantial differences between male and female rats come into view if the observed results are comprehended and visualised as patterns of oxidative stress markers (Fig. 9). Different patterns of the aberrations from normal redox balance in males and females show that different mechanisms of AgNP toxicity prevailed in each sex and organ (Fig. 9). Generally, sex differences in the manifestation of toxins, particularly metals, are probably a result of many physiological processes influenced by different, sex-related physical, physiological and hormonal conditions [47, 76]. The final outcomes of the exposure of a male and female organism to a toxic agent may be a result of significant differences in the defence mechanisms but finally seen only as weakly different. Although much more research is needed to describe the differences in the mechanisms underlying the sex-related response to AgNPs, this research has pointed out that AgNP exposure activated different response mechanisms in male and female rats.

The described differences in the reaction of male and female animals to the low and high dose of AgNPs do not seem to be related to the different distribution and elimination of silver from the animals, as proposed earlier [52, 53]. In this study, males from the LD group accumulated more AgNPs than females but experienced less oxidative damage than females. Still, an important thought arose from the accumulation and oxidative stress investigation: even the tissues with low or statistically insignificant AgNPs accumulation (like liver in LD females and kidneys in LD males) showed signs of the damaging effect of AgNPs. This finding emphasizes the significance of AgNPs accumulation in the exposed organism and the importance of assessment of the toxic effects at low doses.

Assessment of blood parameters in the exposed rats has proven that even low doses of AgNPs and mild disturbance of redox balance may cause functional damage to tissues and organs. Elevated ALP in males exposed to low AgNP dose may be associated with liver damage, as was observed in mice exposed to AgNPs for 14 days showing elevated AST and ALP [27]. This animal group also showed overactivity of CK indicating damages in the heart, skeletal muscle, or brain. Since the AgNPs accumulation in the brain was very high in this animal group, the brain damage cannot be excluded. The CK activity was also elevated in female rats in a dose-dependent manner and it correlated with the AgNPs accumulation in the brain and heart, indicate damages in these organs.

Evaluation of differential blood count revealed that all populations of leukocytes, except MON in males, showed lower numbers in treated animals compared to control animals that may be indicative of bone marrow deficiency [77]. However, RBC and PLT were not affected. Dobrzynska et al. showed previously that the genotoxicity of NPs can vary between different cell types [78]. Reduction of leukocytes, particularly LYM, was more notable in males than in females, showing an obvious difference in the sex-related response to AgNP. It has been shown that gut-associated immune response and expression of genes responsible for gastrointestinal permeability are also affected in a sex-specific manner [54, 55].

## Conclusion

Sub-acute oral exposure to AgNPs induced oxidative stress in the liver and kidneys of female and male Wistar rats. Sex-related differences were noticed for all tested parameters including biodistribution, bioaccumulation, markers of systemic toxicity, and oxidative stress. While female rats eliminated AgNPs more efficiently upon exposure to the lower dose, the opposite was observed for treatment with the higher dose. In the blood, liver, kidney and hearth of females exposed to higher dose, the accumulation was two to three times higher than in males, while in most organs of digestive tract, it was more than ten times higher. The sex-related difference was also observed for Mt genes and protein expression as females did not show any significant changes and males showed a significant reduction compared to control animals. Results obtained for markers of oxidative stress response obtained different patterns of their aberrations in the organs of males and females, indicating different mechanisms of action of accumulated silver in males compared to females, which should be mechanistically investigated. As the final form of accumulated silver was not identified by this study, future efforts should be directed towards elucidation if sex could play the role in the biotransformation of AgNPs during their administration, distribution, metabolism, and excretion.

## Methods

### The aim, design, and setting of the study

This study aimed to clarify the sex differences in the outcomes of sub-acute oral exposure to AgNPs. Wistar rats of both sexes were daily gavaged by low doses (0.1 and 1 mg Ag/kg b.w.) of PVP-coated small-sized (10 nm) AgNPs. After 28 days of exposure, blood and organs of male and female rats were analysed by means of biodistribution and accumulation of Ag, while liver and kidneys were evaluated by ROS and GSH levels, the activity of CAT, SOD, and GPx, expression of Mt genes and levels of Mt proteins. Blood biochemistry and hematological parameters were assessed as indicators of functional damage induced by AgNPs.

### Synthesis and characterisation of silver nanoparticles

Unless otherwise stated, all reagents used for AgNPs synthesis were purchased from the Sigma-Aldrich Chemie GmbH (Munich, Germany) and glassware was cleaned with 10% (v/v) HNO_3_ (Merck Suprapur, Darmstadt, Germany) and rinsed thoroughly with ultrapure water before use. The ultrapure water was prepared by Milli-Q® filtration system (Millipore, Darmstadt, Germany).

The PVP-coated AgNPs were synthesized according to the procedure described previously [79]. Briefly, 0.3% (w/v) PVP solution was prepared by dissolving the appropriate amount of PVP in 190 mL of ultrapure water. Subsequently, 5 mL of 90 mM AgNO_3_ was added and allowed to stir constantly on a magnetic stirrer plate in the ice bath. Finally, 5 mL of 320 mM NaBH_4_ solution was added dropwise (about 1 drop/s) under vigorous stirring. The mixture was left under constant stirring for 45 min in an ice bath and protected from light until obtaining brownish colloid. Freshly prepared AgNPs were centrifuged at 15,000 × *g* for 45 min, resuspended in the ultrapure water, and stored in the dark at 4 °C until use.

Total Ag concentration in AgNPs colloidal suspension was determined in acidified solutions (5% HNO_3_) using the Agilent Technologies 7500cx ICP-MS (Agilent, Waldbronn, Germany).

The size distribution and ζ potential of AgNPs were measured using dynamic light scattering (DLS) and electrophoretic light scattering (ELS) methods, respectively, on the Zetasizer Nano ZS (Malvern, UK) equipped with a green laser (532 nm). Obtained data were processed by the Zetasizer software 6.32 (Malvern instruments). Results of size distribution are reported as volume distributions and represented as an average value of 10 measurements, whereas ζ potential values are expressed as an average of 5 measurements.

A dissolution test was performed in ultrapure water by diluting AgNPs suspensions to the final concentration of 10 mg/L and kept on a shaker for 1 h at room temperature and protected from light. Subsequently, aliquots were taken and subjected to ultrafiltration using Amicon Ultra-4 filters with a cut-off size of 3 KDa (Merck Millipore, Darmstadt, Germany) at the 15,000 × *g* for 30 min. The filtrates were immediately acidified with HNO_3_ to the final acid content of 5% (v/v) and the Ag concentration was determined by the ICP-MS.

Synthesized and purified AgNPs were visualized using TEM (Zeiss 902A) operated in bright field mode at an acceleration voltage of 80 kV. Samples were prepared on a Formvar®coated copper grid by depositing a drop of suspension and air-drying it at room temperature. The primary size was determined from the cross-sectional area of the AgNPs using ImageJ software. Primary particles (in total 100) were distinguished from aggregates by tracing them manually.

### Conditions of animal experiments

Wistar rats of both sexes, aged 12 weeks and weighing 320–350 g body weight (b.w.) for males and 190–220 g b.w. for females, were bred under specific pathogen-free (SPF) conditions at the Animal Breeding Unit, Institute for Medical Research and Occupational Health, Zagreb, Croatia. They were acclimated in the controlled environment (temperature: 23 ± 2 °C; humidity: 55 ± 7% and light: 12 h light/dark cycle) and fed with standard GLP certified food (Mucedola, 4RF21, Italy) and water ad libitum. Rats were randomly assigned to experimental groups to avoid any bias. After treatment with the test substance, animals were sacrificed under general anesthesia using an anaesthetic cocktail (Narketan, Vetoquinol UK Ltd., 80 mg/kg b.w.; Xylapan, Vetoquinol UK Ltd., 12 mg/kg b.w., *i.p*.) in order to avert any pain caused by exsanguinations and tissue harvesting.

All animal experiments were approved by the Institutional Animal Care and Use Committee and were in accordance with the ethical codex for animal welfare of the Croatian Society for Laboratory Animal Science and with international standards.

### Design of animal experiments

Animals were divided into 3 groups (n = 4 per group and per sex): (1) low dose (LD) group treated perorally (*p.o*.) with PVP-coated AgNPs in a daily dose of 0.1 mg Ag/kg b.w.; (2) high dose (HD) group treated *p.o*. with PVP-coated AgNPs in a daily dose of 1 mg Ag/kg b.w.; (3) control (Ctl) group treated *p.o*. by physiological solution on a daily basis. The distribution of animals to the groups was random. The selection of doses and surface coating of AgNPs was based on our previous studies [42]. Following the 28 days of daily oral AgNPs administration, rats were sacrificed under general anaesthesia. All animals were sacrificed at the same day, 24 h after last oral gavage. The intracardiac puncture was used to collect the whole blood in heparinised tubes. After washing in ice-cold physiological saline, organs (liver, kidney, heart, brain, stomach, duodenum, jejunum, ileum, cecum, colon, testis, epididymis, ovaries) were weighed and divided into small pieces. The stomach and gastrointestinal segments were washed from the inside to remove the content. Kidneys were divided into the cortex, outer and inner stripe/papilla after transverse cutting, whereas the liver was divided into small pieces weighing roughly 300 mg. Tissue samples used for the measurement of accumulated AgNPs were immediately frozen at − 20 °C. Fresh tissue homogenates of the liver and kidneys were used to analyse the levels of ROS and GSH, and the activities of CAT, SOD, and GPx. Tissue homogenates were prepared in 0.05 M phosphate buffer solution (PB, pH 7.4) containing 0.1 mM ethylenediaminetetraacetic acid (EDTA) using a motor-driven homogenizer on an ice bath.

### Determination of accumulated silver in organs

The total Ag content in different organs and tissues was measured by the ICP-MS after microwave-assisted acid digestion. An Agilent Technologies 7500cx ICP-MS system (Agilent, Waldbronn, Germany) equipped with an integrated auto-sampler, a Scott Quartz spray chamber and a MicroMist nebulizer (Glass expansion, Australia) was used. According to Agilent Technologies’ recommendations, operating conditions were normal for general and high matrix analysis. The tuning solution (Agilent Technologies, Japan) containing 10 μg/L of Li, Y, Ce, Tl and Co in 2% (w/v) HNO_3_ was used daily in order to achieve satisfying intensities for both oxide ions and doubly charged ones as well as to obtain lower yields. Calibration of ICP-MS system was performed by external standards method, using calibration standards prepared from stock elemental standard solutions of 1000 mg/L from Merck (Darmstadt, Germany) and internal standards of Rh and Lu. “Internal standard stock solution” was added to both samples and standards to achieve the final concentration of 10 µg/L. Before each series of samples, a reagent blank was measured to ensure contamination control. Calibration curves were created linearly after deduction of the reagent blank. Tissue samples were digested in the quartz digestion vessels with the microwave UltraCLAVE IV Milestone digestion device (MLS GmbH Mikrowellen-Laborsysteme, Leutkirch, Germany). Prior to irradiation at 800 W and 120 °C for 10 min, Suprapur 65% HNO3 (Merck, Darmstadt, Germany) was added to the precisely weighed tissue samples, followed by irradiation at 1600 W and 250 °C for 30 min. This resulted in the absolute and almost concurrent dissolution of tissue samples to colourless solutions. The same microwave procedure was used to prepare a set of digestion blanks. Additionally, after cooling the vessels ultrapure water was added to the final volume of 50 mL, which resulted in the overall dilution of 200 v/m and the final solution comprising of 10% v/v HNO_3_. All data are expressed as total Ag concentration, in mg Ag per kg of wet tissue.

### Biochemical analysis

Intracellular ROS in tissue homogenates were measured using 2′,7′-dichlorofluorescin diacetate (DCFH-DA) and dihydroethidium (DHE) staining assays. In the presence of superoxide radical, DHE is oxidized to a fluorescent 2-hydroethidium (EOH), which allows the accurate measurement of DHE fluorescence. Due to the high stability of EOH, there is no risk of interconversion variability [80]. The DCFH-DA conversion into highly fluorescent product 2′,7′-dichlorofluorescein (DCF) happens in two steps. Firstly, DCFH-DA is converted into non-fluorescent 2′,7′-dichlorofluorescin (DCFH) by cellular esterase, which is then oxidized to the fluorescent DCF product in the presence of hydroxyl radical [81].

An ice-cold 40 mM Tris–HCl buffer (pH 7.4) was used for diluting fresh 10% (w/v) tissue homogenates to 0.25% (w/v) homogenates. 0.1 ml of diluted tissue homogenates were pipetted into wells of 96-well plate following the addition of 20 µL 0.12 mM DCFH-DA or DHE. Autofluorescence was examined by preparing the tissue without the addition of a dye. After incubating the samples for 20 min at 37 °C, fluorescence was determined at 488 nm excitation and 525 nm emission wavelengths using a fluorescence plate reader Victor3™ (Perkin-Elmer, United Kingdom).

Monochlorobimane (MBCl) fluorescent probe was used for measuring the GSH level in the liver and kidney homogenates. The reaction of MBCl with GSH is highly specific and results in forming fluorescent adduct [82]. Diluted 0.25% (w/v) tissue homogenates were prepared in the 40 mM Tris–HCl buffer (pH 7.4) and were placed on ice prior to the analysis. Furthermore, 0.1 mL of 0.25% homogenate portions were pipetted into wells of 96-well plate following the addition of 20 µL of 0.24 mM MBCl. Autofluorescence was examined by preparing the tissue without the addition of a fluorescent probe. All samples were incubated for 30 min at 37 °C. Fluorescence was determined at 355 nm excitation and 460 nm emission wavelengths using a Victor3™ plate reader.

SOD activities were analysed using the method described by Marklund and Marklund due to its consistency and reproducibility [83]. Using this method, the effect of ascorbic and uric acid concentrations on the SOD activity in tissues is avoided. Briefly, the mixture of 0.1 mL of 0.25% (w/v) tissue homogenate and 1.9 mL of a solution containing 1 mM of EDTA in 50 mM Tris–HCl buffer (pH 8.2) was placed in a quartz cuvette. Additionally, 0.2 mL of 2.2 mM pyrogallol was added to start the reaction. Using a UV–Vis spectrophotometer (CARY 300, Varian Inc., Australia) oxidation of pyrogallol was followed between 0 and 5 min at 320 nm. The lag of 1.5 min was allowed for the steady-state of autooxidation of pyrogallol to be attained, which was necessary to reach reproducibility. One unit (U) of SOD activity was defined as the amount that reduced the absorbance change by 50%, and results were normalized based on total protein content (U/mg protein).

According to the procedure of Flohe and Gunzler GPx activity in the liver and kidneys was assayed [84]. Briefly, 1 mL of reaction mixture was prepared by addition of 0.4 ml of 100 mM PB (pH 7.4), 0.1 ml of 4 mM GSH, 0.1 ml of 10 mM NaN_3_, 0.1 ml of 1 mM H_2_O_2_, and 0.3 ml of 0.25% (w/v) tissue homogenate following by incubation at 37 °C for 15 min. After the addition of 0.5 ml of 5% trichloroacetic acid, the reaction was terminated and tubes were centrifuged at 1,500 × *g* for 5 min. Subsequently, 0.1 ml of the reaction supernatant was mixed with 0.9 mL of 0.7 mM 5,5′-dithiobis-(2-nitrobenzoic acid) (DTNB) solution prepared in 100 mM PB (pH 7.4) and absorbance at 420 nm was measured. GPx activity was determined using the molar extinction coefficient of 6.22 × 10^3^ M cm^−1^ and was expressed as µ moles of GSH utilized/minute/mg protein at 37 °C. One unit of GPx converts 1 µmol of GSH in 1 min per g of wet tissue.

The activity of CAT in the liver and kidney samples was measured using the previously described method [82]. The reaction mixture consisted of 1.8 mL 50 mM PB (pH 7.4), and 0.1 mL of 0.25% (w/v) tissue homogenates. Prior to absorbance measurement, the reaction was initiated by the addition of 0.1 mL 1 M H_2_O_2_. Changes in the absorbance were measured at 240 nm for 60 s at room temperature. The CAT activity was expressed as the unit defined as mmol of H_2_O_2_ consumed per min per gram of wet tissue. One unit of CAT activity is the amount of enzyme that liberates 50% of the peroxide oxygen from the H_2_O_2_ solution of any concentration in 60 s.

### Blood analysis

Blood samples were collected by jugular venipuncture, using the Vacutainer blood collection system (Becton, Dickinson and Co., Rutherford, NJ). Serum was separated by centrifugation at 1000 × *g* at 4 °C for 15 min, within 1 h of collection, and aliquots were stored at − 80 °C until analysis.

The haematological parameters in whole blood (HGB, MCH, MCHC, MCV, HCT, RBC, RDW, WBC, MON, GRA, LYM, PLT, and MPV were analysed on the Abbott Cell-Dyn CD 3500 automated haematology analyser (Abbott Diagnostic division, Mountain View, CA).

Serum triglycerides, total cholesterol, and creatinine levels, as well as the activities of ALT, AST, ALP, CK, and LDH, were determined by standard commercial reagent packages (Beckman Coulter Biomedical Ltd., O’Callaghans Mills, Ireland) with the Beckman Coulter AU 680 biochemical analyser (Beckman Coulter Biomedical Ltd. München, Germany).

### RNA isolation and gene expression analysis

Total RNA was extracted from the RNAlater-stored (Sigma-Aldrich Chemie GmbH, Munich, Germany) liver and kidney tissue pieces using the IllustraTM RNAspin Mini RNA Isolation Kit (GE Healthcare, Amersham, UK) according to the product manual and stored at − 80 °C until analyses. The procedure included the DNA digestion step. Quantity and purity of the extracted RNA were determined using the NanoDrop spectrophotometer (Thermo Fisher Scientific, Wilmington, DE, USA), and the integrity was confirmed by formaldehyde-agarose-gel electrophoresis. Reverse transcription of total RNA was performed using High-Capacity cDNA Reverse Transcription Kit (Applied Biosystems, Foster City, CA) with 1 μg of RNA per 20 μL reverse transcription reaction, according to the manufacturer's instructions. cDNA corresponding to 50 ng RNA was amplified for 40 cycles in a 25 μL PCR mix (TaqMan™ Gene Expression Master Mix, Applied Biosystems, Foster City, CA) that contained 1xTaqMan primers/probe mix, on 7300 Real-Time PCR System (Applied Biosystems, Foster City, CA). 2xTaqMan primers/probe mixes for mRNA of interest, as well as for reference gene, β-actin (Actb), were available as inventoried validated assays (assay ID: Rn01536930_g1 for Mt1a, Rn01536588_g1 for Mt2a, Rn00588658_g1 for Mt3 and Rn00667869_m1 for Actb), and cycling conditions were standard conditions recommended by the manufacturer for applied PCR mix and TaqMan primers/probes. The absence of potential gDNA contamination was assessed by the inclusion of RNA isolate as a template control and the absence of PCR-product/cDNA contamination was assessed by the inclusion of a no template control (NTC) in every run. All reactions were carried out in triplicates. The calculation of relative quantities i.e. relative mRNA levels of target genes was performed based on the standard delta-delta-Ct method as recommended in Hellemans et al. [85], which includes normalization against reference gene Actb.

### Western-blotting analysis

SDS–polyacrylamide gel electrophoresis was performed in 0.75 mm thick 12% polyacrylamide gels (acrylamide/bisacrylamide 29:1) after stacking in 3% gel. As standard, Page Ruler Plus Prestained Protein Ladder (Thermo Scientific) was applied. Separated proteins (10 µg/well) were transferred onto a PVDF-membrane using the semi-dry transfer technique and fixed in 1% glutaraldehyde/H_2_O. Blocking was performed in 5% non-fat dry milk/TBST for 2 h. Incubation with primary antibody was performed overnight, and, after washing, with secondary horseradish-peroxidase (HRP)-labelled antibody for 1 h. Visualisation using Immobilon Western Chemiluminescent HRP substrate (Millipore) was recorded on Amersham™ Imager 600 (GE Healthcare, Uppsala, Sweden). The first round of detection was followed by stripping for 45 min at 50 °C in stripping solution (100 mM 2-mercaptoethanol, 2% (w/v) SDS, 62.5 mM Tris–HCl, pH 6.7). After washing, the blocking step was the first in the next round of immunodetection performed as described above. Primary antibodies were mouse monoclonal anti-metallothionein MT1/2 (M0639, Dako, 1:1 000) and mouse anti-actin monoclonal IgG1kappa (MAB1501, Chemicon®international, 1:1000), and secondary antibody was goat-polyclonal anti-mouse IgG labelled with HRP (ab6789, Abcam, 1:20 000). Quantification of the band intensity was performed as recommended in Gassmann et al. [86], using the ImageJ 1.50 g software (Windows version of NIH Image, https://imagej.nih.gov/ij/index.html), and includes normalization against reference protein actin.

### Statistical analysis

Statistical analysis was performed according to recommendations published previously by Marusteri and Bacera [87]. As data were not normally distributed and more than 2 groups were compared, we performed non-parametric Kruskal–Wallis one-way ANOVA test using the Statistica Software 13.5.0.17 (TIBCO Software Inc., Palo Alto, USA). Data are represented as mean values obtained from four animals including standard deviations (SD). Significant differences (*p* < 0.05 and *p* < 0.005) between controls and treated animals, lower and high dose, respectively, are indicated by the asterisks (* and **, respectively). Significant differences (*p* < 0.05) between animals from the LD and HD groups of the same sex are indicated with hashtags (#), while significant differences (*p* < 0.05) between males and females are denoted with section signs (§).

## Supplementary Information

**Additional file 1.** Title of data: Numerical results.Data description: Tables showing data and statistics of the results of determination of biodistribution and bioaccumulation of AgNPs, biochemical analyses of the liver, kidneys and blood parameters, and Mt mRNA and protein analyses.

## Data Availability

The dataset supporting the conclusions of this article is included within the article (and its additional file).

## Abbreviations

Actb: β-Actin
ADME: Absorption, distribution, metabolism, elimination
AgNPs: Silver nanoparticles
ALP: Alkaline phosphatase
ALT: Alanine transaminase
AST: Aspartate transaminase
CAT: Catalase
CK: Creatine kinase
Ctl: Control
DCF: 2′,7′-Dichlorofluorescein
DCFH-DA: 2′,7′-Dichlorofluorescin diacetate
DHE: Dihydroethidium
DLS: Dynamic light scattering
DTNB: 5,5′-Dithiobis-(2-nitrobenzoic acid)
EDTA: Ethylenediaminetetraacetic acid
ELS: Electrophoretic light scattering
EOH: 2-Hydroethidium
GPx: Glutathione peroxidase
GRA: Granulocytes
GSH: Glutathione
HCT: Hematocrit
HD: High dose (1 mg Ag/kg b.w.) of AgNPs
HGB: Hemoglobin
ICP-MS: Inductively coupled plasma mass spectrometer
LD: Low dose (0.1 mg Ag/kg b.w.) of AgNPs
LDH: Lactate dehydrogenase
LYM: Lymphocyte
MBCl: Monochlorobimane
MCH: Mean corpuscular hemoglobin
MCHC: Mean corpuscular hemoglobin concentration
MCV: Mean corpuscular volume
MON: Monocyte
MPV: Mean platelet volume
MT: Metallothionein
PB: Phosphate buffer
PLT: Platelets
PVP: Polyvinylpyrrolidone
RBC: Red blood cells
RDW: Red cell distribution width
ROS: Reactive oxygen species
SOD: Superoxide dismutase
SD: Standard deviation
TEM: Transmission electron microscopy
WBC: White blood cell
