# Supplementary material for: Sex affects the response of Wistar rats to polyvinyl pyrrolidone (PVP)-coated silver nanoparticles in an oral 28 days repeated dose toxicity study
Source: Part Fibre Toxicol. 2021 Oct 18;18:38. doi: 10.1186/s12989-021-00425-y (PMC8522010; doi:10.1186/s12989-021-00425-y)
Supplement: Supplementary file 1 — Additional file 1. Title of data: Numerical results.Data description: Tables showing data and statistics of the results of determination of biodistribution and bioaccumulation of AgNPs, biochemical analyses of the liver, kidneys and blood parameters, and Mt mRNA and protein analyses. [file 12989_2021_425_MOESM1_ESM.docx]

**Additional file 1: Numerical results**

**Sex affects the response of Wistar rats to polyvinyl pyrrolidone (PVP)-coated silver nanoparticles in an oral 28 days repeated dose toxicity study**

Marija Ćurlin^^[[1]](#footnote-1)^,*^, Rinea Barbir^2^, Sanja Dabelić^3^, Marija Ljubojević^2^, Walter Goessler^5^, Vedran Micek^2^, Irena Žuntar^3^, Mirela Pavić^4^, Lucija Božičević^2^, Ivan Pavičić^2^, Ivana Vinković Vrček^2,*^

*^1^School of Medicine, University of Zagreb, Šalata 3, 10 000 Zagreb, Croatia*

*^2^Institute for Medical Research and Occupational Health, Ksaverska cesta 2, 10 000 Zagreb, Croatia*

*^3^Faculty of Pharmacy and Biochemistry, University of Zagreb, Ante Kovačića 1, 10 000 Zagreb, Croatia*

*^4^Faculty of Veterinary Medicine, University of Zagreb, Heinzelova 55, 10 000 Zagreb, Croatia*

*^5^Institute of Chemistry, University of Graz, Universitätsplatz 1/1, 8 010 Graz, Austria*

**Table 1.** Peroxy radical levels measured by DCFH-DA assay, superoxide radical levels measured by DHE assay and levels of glutathione given as % of fluorescence compared to control animals in the liver and kidney of male and female Wistar rats after the 28-day oral administration of low dose (LD = 0.1 mg per kg b.w.) and high dose (HD = 1 mg per kg b.w.) of silver nanoparticles (AgNPs) stabilized with polyvinylpyrrolidone (PVP-AgNPs). Results are expressed in % as the mean values obtained from organs of four animals ± standard deviations. Significant differences (*p* < 0.05 and *p* < 0.001) between controls and treated animals (for both LD and HD groups) are indicated by the asterisks (* and **, respectively). Significant differences (*p* < 0.05) between LD and HD groups of the same sex are labelled with hashtags (^#^) and significant differences (*p* < 0.05) between males and females with section signs (^§^).

| **Tissue** | **% of fluorescence compared to Ctl** | **Males** | | | **Females** | | |
| --- | --- | --- | --- | --- | --- | --- | --- |
|  |  | **Ctl** | **LD** | **HD** | **Ctl** | **LD** | **HD** |
| **Liver** | **Peroxy radical** | 100 ± 1.72 | 57.15 ± 1.56* | 179.63 ± 13.06* | 100 ± 2.02 | 148.12 ± 4.59**^§^ | 89.13 ± 1.76*^#§^ |
|  | **Superoxide radical** | 100 ± 3.49 | 104.40 ± 0.71 | 371.67 ± 31.28**^#^ | 100 ± 1.97 | 90.62 ± 0.96*^§^ | 198.31 ± 17.58**^#^ |
|  | **GSH** | 100 ± 0.89 | 87.38 ± 1.24** | 91.99 ± 1.09** | 100 ± 1.14 | 88.95 ± 1.54** | 107.03 ± 1.33^#§^ |
| **Kidney** | **Peroxy radical** | 100 ± 1.55 | 49.41 ± 1.16** | 55.38 ± 1.15* | 100 ± 1.97 | 113.74 ± 2.31^§^ | 177.54 ± 9.17*^#§^ |
|  | **Superoxide radical** | 100 ± 1.17 | 68.52 ± 1.05** | 70.11 ± 0.81** | 100 ± 1.63 | 86.63 ± 1.40**^§^ | 90.89 ± 1.60*^§^ |
|  | **GSH** | 100 ± 3.56 | 119.06 ± 2.24* | 129.62 ± 3.52** | 100 ± 1.44 | 83.63 ± 3.15*^§^ | 75.55 ± 1.53**^§^ |

**Table 2.** SOD activity, CAT activity and GPx activity given as units of enzyme activity per gram of wet tissue in the liver and kidney of male and female Wistar rats after the 28-day oral administration of low dose (LD = 0.1 mg per kg b.w.) and high dose (HD = 1 mg per kg b.w.) of silver nanoparticles (AgNPs) stabilized with polyvinylpyrrolidone (PVP-AgNPs). Results are expressed as the mean values obtained from organs of four animals +/- standard deviations. Significant differences (*p* < 0.05 and *p* < 0.001) between controls and treated animals (for both LD and HD groups) are indicated by the asterisks (* and **, respectively). Significant differences (*p* < 0.05) between LD and HD groups of the same sex are labelled with hashtags (^#^) and significant differences (*p* < 0.05) between males and females with section signs (^§^).

| **Tissue** | **Enzyme** (U/g wet tissue) | **Males** | | | **Females** | | |
| --- | --- | --- | --- | --- | --- | --- | --- |
|  |  | **Ctl** | **LD** | **HD** | **Ctl** | **LD** | **HD** |
| **Liver** | **SOD** | 49.71 ± 3.26 | 50.78 ± 2.09 | 59.34 ± 2.34 | 105.43 ± 3.05 | 72.45 ± 4.84* | 107.92 ± 4.30^#§^ |
|  | **CAT** | 69.19 ± 3.68 | 52.31 ± 1.43** | 66.37 ± 1.29^#^ | 27.09 ± 1.18 | 43.61 ± 1.72** | 43.13 ± 1.60*^§^ |
|  | **GPx** | 69.84 ± 4.46 | 56.46 ± 4.28 | 64.06 ± 1.88 | 50.41 ± 0.66 | 37.82 ± 4.68 | 37.78 ± 3.00* |
| **Kidney** | **SOD** | 86.94 ± 2.38 | 66.81 ± 1.60 | 49.84 ± 1.64**^#^ | 67.32 ± 1.35 | 25.68 ± 2.44**^§^ | 65.97 ± 2.47^#§^ |
|  | **CAT** | 17.03 ± 1.11 | 15.97 ± 0.73 | 10.14 ± 0.25**^#^ | 13.27 ± 0.65 | 17.92 ± 0.75 | 7.84 ± 0.41*^#^ |
|  | **GPx** | 14.68 ± 1.92 | 16.91 ± 1.00 | 0.84 ± 0.42^#^ | 12.52 ± 1.52 | 17.45 ± 0.59 | 7.97 ± 3.67^#^ |

**Table 3.** mRNA expression of metallothionein genes Mt1a and Mt2a in the liver and Mt1a, Mt2a, and Mt3 in kidneys of male and female Wistar rats after 28-day oral exposure to low (LD = 0.1 mg per kg b.w.) and high dose (HD = 1 mg per kg b.w.) of PVP-AgNPs. mRNA expression levels are presented as relative values compared to mRNA levels of control samples. All results are expressed as the mean values obtained from organs of four animals ± standard deviations. Significant differences (*p* < 0.05) between controls and treated animals (for both LD and HD groups) are indicated by the asterisks (*). Significant differences (*p* < 0.05) between males and females are indicated with section signs (^§^). Differences between LD and HD groups of the same sex were not significant (*p* > 0.05).

| **Tissue** | **Mt gene** | **Males** | | | **Females** | | |
| --- | --- | --- | --- | --- | --- | --- | --- |
|  |  | **Ctl** | **LD** | **HD** | **Ctl** | **LD** | **HD** |
| **Liver** | **Mt1a** | 1.00 ± 0.38 | 0.37 ± 0.20 | 0.55 ± 0.14 | 1.00 ± 0.10 | 1.64 ± 0.62^§^ | 1.30 ± 0.18 |
|  | **Mt2a** | 1.00 ± 0.20 | 0.43 ± 0.22 | 0.54 ± 0.17 | 1.00 ± 0.18 | 1.24 ± 0.45 | 1.19 ± 0.31 |
| **Kidney** | **Mt1a** | 1.00 ± 0.19 | 0.48 ± 0.13 | 0.59 ± 0.12 | 1.00 ± 0.07 | 0.82 ± 0.11 | 1.07 ± 0.11 |
|  | **Mt2a** | 1.00 ± 0.08 | 0.71 ± 0.08 | 0.57 ± 0.10* | 1.00 ± 0.10 | 1.03 ± 0.31 | 1.25 ± 0.24^§^ |
|  | **Mt3a** | 1.00 ± 0.03 | 0.66 ± 0.01 | 0.61 ± 0.05* | 1.00 ± 0.12 | 1.00 ± 0.08 | 0.99 ± 0.21 |

**Table 4.** Protein expression of two multimeric forms (~42 kDa and ~24 kDa) of metallothionein MT1/2 proteins, in the liver and kidney of male and female Wistar rats after 28-day oral administration of low (LD = 0.1 mg per kg b.w.) and high dose (HD = 1 mg per kg b.w.) of PVP-AgNPs. Protein expression levels are presented as relative values compared to protein levels of control samples. All results are expressed as the mean values obtained from organs of four animals ± standard deviations. Significant differences (*p* < 0.05) between controls (Ctl) and treated animals (for both LD and HD groups) are indicated by the asterisks (*).

| **Tissue** | **Form of MT1/2** | **Males** | | | **Females** | | |
| --- | --- | --- | --- | --- | --- | --- | --- |
|  |  | **Ctl** | **LD** | **HD** | **Ctl** | **LD** | **HD** |
| **Liver** | **42 kDa** | 1.00 ± 0.34 | 0.52 ± 0.37 | 0.58 ± 0.11 | 1.00 ± 0.28 | 0.68 ± 0.22 | 0.96 ± 0.48 |
|  | **24 kDa** | 1.00 ± 0.24 | 0.39 ± 0.11* | 0.45 ± 0.10 | 1.00 ± 0.20 | 0.93 ± 0.21 | 1.23 ± 0.21 |
| **Kidney** | **42 kDa** | 1.00 ± 0.10 | 0.23 ± 0.11* | 0.21 ± 0.10* | 1.00 ± 0.15 | 0.78 ± 0.22 | 0.80 ± 0.31 |
|  | **24 kDa** | 1.00 ± 0.08 | 0.36 ± 0.15* | 0.33 ± 0.10** | 1.00 ± 0.13 | 0.77 ± 0.24 | 0.86 ± 0.29 |

**Table 5.** Biochemical blood parameters of male and female Wistar rats after 28-day oral administration of low dose (LD = 0.1 mg per kg b.w.) and high dose (HD = 1 mg per kg b.w.) of PVP-AgNPs. All results are expressed as the mean values obtained from organs of four animals ± standard deviations. Differences between controls (Ctl) and treated animals (for both LD and HD groups), differences between males and females and differences between LD and HD groups of the same sex were not significant (*p* > 0.05).

| **Blood parameter** | **Males** | | | **Females** | | |
| --- | --- | --- | --- | --- | --- | --- |
|  | **Ctl** | **LD** | **HD** | **Ctl** | **LD** | **HD** |
| **Creatinine** (µmol/L) | 38.00 ± 2.83 | 41.00 ± 0.82 | 39.50 ± 2.38 | 39.00 ± 2.83 | 40.00 ± 2.71 | 36.75 ± 2.63 |
| **AST** (U/L) | 158.50 ± 21.92 | 199.25 ± 51.23 | 180.75 ± 27.29 | 183.00 ± 25.46 | 136.00 ± 18.57 | 160.50 ± 61.07 |
| **ALT** (U/L) | 36.50 ± 2.12 | 41.75 ± 7.72 | 44.75 ± 10.90 | 43.50 ± 0.71 | 38.75 ± 3.30 | 39.75 ± 9.07 |
| **ALP** (U/L) | 173.50 ± 14.85 | 248.00 ± 30.74 | 216.75 ± 60.40 | 140.50 ± 24.75 | 139.50 ± 25.41 | 153.00 ± 18.57 |
| **CK** (U/L) | 6252.00 ± 1462.30 | 8326.25 ± 4479.92 | 6551.75 ± 3345.56 | 3361.00 ± 511.95 | 4777.50 ± 911.11 | 5824.50 ± 2822.06 |
| **LDH** (U/L) | 1801.50 ± 225.57 | 2286.25 ± 764.02 | 1960.50 ± 374.29 | 1860.50 ± 334.46 | 1045.25 ± 321.65 | 1082.50 ± 451.07 |
| **Triglycerides** (mmol/L) | 1.30 ± 0.28 | 1.33 ± 0.42 | 1.40 ± 0.24 | 0.70 ± 0.28 | 0.75 ± 0.30 | 0.73 ± 0.22 |
| **Cholesterol** (mmol/L) | 1.45 ± 0.07 | 1.30 ± 0.12 | 1.33 ± 0.17 | 1.05 ± 0.07 | 1.30 ± 0.14 | 1.10 ± 0.14 |

**Table 6.** Hematological parameters of male and female Wistar rats after 28-day oral exposure to low dose (0.1 mg per kg b.w.) and high dose (1 mg per kg b.w.) of PVP-AgNPs. All results are expressed as the mean values obtained from organs of four animals ± standard deviations. Significant differences (*p* < 0.05) between LD and HD groups of the same sex are labelled with hashtags (^#^). Differences between controls (Ctl) and treated animals (for both LD and HD groups) and differences between males and females were not significant (*p* > 0.05).

| **Hematological parameter** | **Males** | | | **Females** | | |
| --- | --- | --- | --- | --- | --- | --- |
|  | **Ctl** | **LD** | **HD** | **Ctl** | **LD** | **HD** |
| **WBC** (10^9^/L) | 7.85 ± 0.21 | 5.90 ± 1.21 | 4.53 ± 0.06 | 5.55 ± 0.07 | 3.67 ± 0.98 | 4.10 ± 0.57 |
| **RBC** (10^12^/L) | 7.23 ± 0.10 | 8.07 ± 0.59 | 7.96 ± 0.54 | 6.99 ± 0.72 | 6.46 ± 0.17 | 7.08 ± 0.56 |
| **HGB** (g/L) | 152.50 ± 13.44 | 160.00 ± 6.56 | 158.67 ± 5.03 | 155.50 ± 2.12 | 144.67 ± 4.04 | 147.75 ± 8.81 |
| **HCT** (L/L) | 0.38 ± 0.02 | 0.42 ± 0.03 | 0.41 ± 0.02 | 0.37 ± 0.03 | 0.35 ± 0.01 | 0.38 ± 0.03 |
| **PLT** (10^9^/L) | 822.00 ± 110.31 | 835.33 ± 79.20 | 603.00 ± 370.84 | 825.00 ± 106.07 | 765.33 ± 73.79 | 876.75 ± 46.50 |
| **MCV** (fL) | 53.00 ± 2.83 | 52.33 ± 1.15 | 51.33 ± 1.15 | 53.50 ± 2.12 | 54.00 ± 1.00 | 53.25 ± 0.96 |
| **MCH** (pg) | 21.05 ± 1.48 | 19.93 ± 1.16 | 19.27 ± 0.67 | 22.35 ± 2.05 | 22.40 ± 0.82 | 20.93 ± 0.64 |
| **MCHC** (g/L) | 397.50 ± 7.78 | 382.00 ± 17.44 | 375.67 ± 11.93 | 417.00 ± 25.46 | 415.33 ± 9.07 | 392.00 ± 11.80 |
| **RDW** (L%) | 14.40 ± 1.27 | 14.03 ± 1.02 | 12.87 ± 0.15 | 12.90 ± 1.41 | 14.27 ± 0.90 | 12.78 ± 0.78 |
| **MPV** (fL) | 7.25 ± 0.49 | 7.77 ± 1.01 | 6.97 ± 0.06 | 7.30 ± 0.28 | 7.93 ± 0.21 | 6.73 ± 0.15^#^ |
| **LYM** (10^9^/L) | 5.05 ± 0.07 | 3.67 ± 0.68 | 2.83 ± 0.32 | 3.35 ± 0.49 | 2.07 ± 0.81 | 2.75 ± 0.39 |
| **MON** (10^9^/L) | 0.60 ± 0.14 | 0.37 ± 0.06 | 0.33 ± 0.06 | 0.40 ± 0.00 | 0.23 ± 0.0 | 1.10 ± 0.06 |
| **GRA** (10^9^/L) | 2.20 ± 0.14 | 1.87 ± 0.72 | 1.37 ± 0.32 | 1.80 ± 0.42 | 1.37 ± 0.12 | 1.10 ± 0.27 |

1. * Corresponding author at School of Medicine, University of Zagreb: Marija Ćurlin; e-mail: [marija.curlin@mef.hr](mailto:marija.curlin@mef.hr); Corresponding author at Institute for Medical Research and Occupational Health: Ivana Vinković Vrček; e-mail: ivinkovic@imi.hr [↑](#footnote-ref-1)
